# The Orphan Cytokine Receptor CRLF3 Emerged With the Origin of the Nervous System and Is a Neuroprotective Erythropoietin Receptor in Locusts

**DOI:** 10.3389/fnmol.2019.00251

**Published:** 2019-10-11

**Authors:** Nina Hahn, Luca Büschgens, Nicola Schwedhelm-Domeyer, Sarah Bank, Bart R. H. Geurten, Pia Neugebauer, Bita Massih, Martin C. Göpfert, Ralf Heinrich

**Affiliations:** ^1^Department of Cellular Neurobiology, Institute for Zoology and Anthropology, Georg-August University of Göttingen, Göttingen, Germany; ^2^Department of Animal Evolution and Biodiversity, Institute for Zoology & Anthropology, Georg-August University of Göttingen, Göttingen, Germany

**Keywords:** neuroprotection, erythropoietin, cytokine receptor, *Locusta migratoria*, soaking RNA interference, nervous system, ancient receptor, orphan receptor

## Abstract

The orphan cytokine receptor-like factor 3 (CRLF3) was identified as a neuroprotective erythropoietin receptor in locust neurons and emerged with the evolution of the eumetazoan nervous system. Human CRLF3 belongs to class I helical cytokine receptors that mediate pleiotropic cellular reactions to injury and diverse physiological challenges. It is expressed in various tissues including the central nervous system but its ligand remains unidentified. A CRLF3 ortholog in the holometabolous beetle *Tribolium castaneum* was recently shown to induce anti-apoptotic mechanisms upon stimulation with human recombinant erythropoietin. To test the hypothesis that CRLF3 represents an ancient cell-protective receptor for erythropoietin-like cytokines, we investigated its presence across metazoan species. Furthermore, we examined CRLF3 expression and function in the hemimetabolous insect *Locusta migratoria*. Phylogenetic analysis of CRLF3 sequences indicated that CRLF3 is absent in Porifera, Placozoa and Ctenophora, all lacking the traditional nervous system. However, it is present in all major eumetazoan groups ranging from cnidarians over protostomians to mammals. The CRLF3 sequence is highly conserved and abundant amongst vertebrates. In contrast, relatively few invertebrates express CRLF3 and these sequences show greater variability, suggesting frequent loss due to low functional importance. In *L. migratoria*, we identified the transcript *Lm-crlf3* by RACE-PCR and detected its expression in locust brain, skeletal muscle and hemocytes. These findings correspond to the ubiquitous expression of *crlf3* in mammalian tissues. We demonstrate that the sole addition of double-stranded RNA to the culture medium (called soaking RNA interference) specifically interferes with protein expression in locust primary brain cell cultures. This technique was used to knock down *Lm-crlf3* expression and to abolish its physiological function. We confirmed that recombinant human erythropoietin rescues locust brain neurons from hypoxia-induced apoptosis and showed that this neuroprotective effect is absent after knocking down *Lm-crlf3.* Our results affirm the erythropoietin-induced neuroprotective function of CRLF3 in a second insect species from a different taxonomic group. They suggest that the phylogenetically conserved CRLF3 receptor may function as a cell protective receptor for erythropoietin or a structurally related cytokine also in other animals including vertebrate and mammalian species.

## Introduction

The cytokine receptor-like factor 3 (CRLF3) is a largely uncharacterized orphan cytokine receptor with unknown function and endogenous ligand. The human *crlf3* gene (NCBI Accession No. NM_015986.4, synonyms *Creme9*, *Cytor4*, *p48.2*, *p48.6*) is located on chromosome 17 and spans 2873 base pairs. The human CRLF3 spans 442 amino acids comprising the conserved cytokine receptor motif WSXWS, a single transmembrane segment and an intracellular Janus kinase (JAK) docking site. These characteristics identify CRLF3 as a member of the group 1 in the prototypic class I cytokine receptors that typically bind class 1 helical cytokines (Boulay et al., 2003; Liongue and Ward, 2007). Group 1 also contains the classical erythropoietin receptor (EpoR), the thrombopoietin receptor, the prolactin receptor and the growth hormone receptor. They typically function as homo- or hetero-dimers or associate to multimeric receptor complexes (Boulay et al., 2003). CRLF3 is expressed in various human tissues, including pancreas, kidney, and brain amongst others (Yang et al., 2009). In addition, freshly isolated tumor tissues and some tumor cell lines show elevated CRLF3 expression (Dang et al., 2006; Yang et al., 2009). CRLF3 has been associated with signal transducer and activator of transcription 3 (STAT3) activation, cell cycle regulation, neuronal morphology, and amyotrophic lateral sclerosis (Yang et al., 2009; Hashimoto et al., 2012; Cirulli et al., 2015). However, its physiological function is yet to be determined. Given its structural similarities to EpoR, we have investigated the potential involvement of CRLF3 in erythropoietin (Epo)-mediated neuroprotection.

Even though *epo* and *epoR* are not present in invertebrate genomes, previous *in vitro* and *in vivo* studies demonstrated neuroprotective and neuroregenerative effects of recombinant human erythropoietin (rhEpo) in the insects *Locusta migratoria* and *Tribolium castaneum* (Ostrowski et al., 2011; Miljus et al., 2014; Hahn et al., 2017). We found several parallels between Epo-mediated neuroprotection in mammals and insects including activation of JAK/STAT intracellular signaling, induction of anti-apoptotic proteins, initiation of receptor endocytosis after Epo-binding, and sensitivity to the non-erythropoietic human Epo splice variant EV-3 (Miljus et al., 2014, 2017; Hahn et al., 2017; Heinrich et al., 2017). These findings supported the hypothesis (Brines and Cerami, 2005; Ostrowski et al., 2011; Ghezzi and Conklin, 2013) that Epo signaling originally functioned as an adaptation mechanism to challenging physiological conditions (e.g., infections, metabolic stress, injury, hypoxia) and only later evolved to regulate vertebrate red blood cell production (summarized by Jelkmann, 2011). Since cytokines commonly activate different receptors and cytokine receptors often respond to several cytokine ligands, we explored the hypothesis that CRLF3 serves as the neuroprotective receptor stimulated by rhEpo in insects.

We previously demonstrated that CRLF3 is crucial for Epo-mediated neuroprotection in hypoxia-exposed neurons from the beetle *T. castaneum* (Hahn et al., 2017). In contrast, Epo showed no cell protective effects in *in vitro* studies using macrophage-like Schneider (S2) cells and neuron-like BG2-c2 cells derived from the fruit fly *Drosophila melanogaster* (unpublished data). *Drosophila* lacks a *crlf3* gene (Wyder et al., 2007; Hahn et al., 2017, this study) supporting the hypothesis that CRLF3 may function as a neuroprotective receptor for Epo. Investigating CRLF3 as a neuroprotective Epo receptor contributes to the understanding of Epo as a neuroprotective agent and may support the development of alternative, safe treatments for neurological and neurodegenerative diseases that, unlike Epo itself, do not stimulate adverse side effects (Leist et al., 2004; Unger et al., 2010).

To the present, studies on CRLF3 involvement in Epo-mediated cell protection have only been performed in the beetle *T. castaneum* (Coleoptera). Here, we study locust primary brain cells (*L. migratoria*, Orthoptera) to confirm the hypothesis that CRLF3 represents an evolutionary ancient cell protective receptor. *In vivo* cellular functions can best be modeled *in vitro* by primary cell cultures, since their cellular development took place in natural environment. *In vitro* loss of function studies with mammalian cells require electroporation, lipid-mediated or viral-mediated transfection in order to induce gene targeted RNA interference (RNAi) and are prone to low efficiency. Locust primary brain neurons have the advantage that they spontaneously take up double-stranded RNA from the medium. This initiates the RNAi machinery, specifically suppressing the production of a protein of interest (called soaking RNAi). We applied soaking RNAi for a loss of function study in order to investigate the function of *Lm-*CRLF3 in primary locust brain cells.

The present study provides further evidence for the importance of CRLF3 in Epo-mediated neuroprotection using locust neurons. Moreover, we show *Lm-crlf3* expression in a variety of locust tissues, arguing for a general cell protective function of CRLF3. Phylogenetic analysis resulted in 293 eumetazoan species expressing CRLF3 with the earliest appearance in the last common ancestor of Cnidaria and Bilateria. This indicates that its original function might have been related to the eumetazoan nervous system. Later in evolution, CRLF3 was coopted for functions also in other tissues leading to frequent expression and high sequence conservation amongst vertebrate species. We furthermore validate soaking RNAi in locust neurons as an appropriate technique for robust loss of function studies *in vitro*.

## Materials and Methods

### Primers

### Plasmids

The plasmid pDsRed (GB0100) was a gift from Diego Orzaez (Addgene plasmid # 68202^1^; RRID:Addgene_68202) (Sarrion-Perdigones et al., 2013). *Lm-crlf3* fragment 1 and fragment 2 were designed as two non-overlapping fragments. They were inserted into the pCRII vector (TA Cloning^®^ Kit Dual Promoter with pCR^TM^II vector, Thermo Fisher Scientific, Germany) by TA-cloning, respectively. Then, pCRII_*Lm-crlf3*_F1 and pCRII_*Lm-crlf3*_F2 plasmids were transformed into XL1-Blue competent cells (#200249, Agilent, United States) and purified with the NucleoBond^®^ Xtra Midi kit (Macherey-Nagel, Germany) according to the user manual. The plasmid DNA was eluted in 500 μl H_2_O. *Lm-rpt3* was identified using BLAST and the LocustBase official gene set (OGS CDS V2.4.1)^2^ (Altschul et al., 1990). The sequence LOCMI02241 was determined as *Lm*-rpt3: the full-length CDS (submitted to GenBank with Accession No. MN245517) and a fragment of *Lm-rpt3* were cloned into the pCRII vector as described above. All sequences are summarized in Supplementary File S1.

### Animals

Locusts (*L. migratoria*) were purchased from Feeders & more (Au i.d. Hallertau, Germany) and HW-Terra (Herzogenaurach, Germany). They were kept in groups at 24°C, 55% air humidity and 12 h/12 h dark/light cycle for up to 1 week. Food was composed of organic lettuce leafs and reed *ad libitum*. Since this study was conducted exclusively with insects, it does not require a special permission. All experiments comply with the German laws for animal welfare (“Deutsches Tierschutzgesetz”).

### Phylogenetic Analysis

We searched for CRLF3 sequences with the blastp algorithm and default settings using the Geneious^®^ 11.1.5 (Biomatters, Ltd.) BLAST tool and the human CRLF3 sequence (Q8IUI8.2) as query sequence. The NCBI accession numbers of the resulting hits are listed in Supplementary File S2. The CRLF3 sequence of *L. migratoria* was obtained by RACE PCR (rapid amplification of cDNA-ends with polymerase chain reaction) and submitted to GenBank (Accession No. MN245516). The CRLF3 sequence of *Gryllus bimaculatus* was obtained by using the tblastn search on the ASGARD data base^3^. The resulting hit GB-isotig00932 was translated into all possible reading frames. We used the translated sequence of reverse frame 3 since it comprises the CRLF3 characteristic motif WSXWS and an appropriate stop codon. All amino acid sequences were aligned using ClustalW version 2.1 implemented in Geneious^®^ with default settings. Subsequently, we removed all columns that consisted of more than 50% missing data from the alignment resulting in a length of 438 amino acids. The phylogenetic tree was inferred with IQ-TREE version 1.6.8 (Nguyen et al., 2015) using the suggested substitution model JTT + R6 (Wong et al., 2017). Support values were computed using the implemented ultrafast bootstrap approximation and 1000 replicates (Minh et al., 2013; Hoang et al., 2018). The tree was rooted with the Cnidaria cluster.

### First Strand cDNA Synthesis and RACE PCR

First-strand cDNA was synthesized from 1 μg total RNA of brain tissue using SMARTer^®^ RACE 5′/3′ Kit (Clontech, Takara, France) according to the user manual. Subsequently 5′- and 3′-rapid amplification of cDNA ends (5′ and 3′ RACE) was performed. The respective primers are summarized in Table 1. Gene-specific primers were designed on the partial sequences available at LocustBase^2^ and i5k^4^. The RACE PCR was performed with the following touchdown program and Advantage^®^ 2 Polymerase Mix (Takara, France): initial step at 94°C for 2 min, 5 cycles at 94°C for 30 s and 72°C for 5 min, 10 cycles at 94°C for 30 s, 70°C for 30 s and 72°C for 5 min, 25 cycles at 94°C for 30 s, 68°C for 30 s and 72°C for 5 min, and a final step at 72°C for 5 min. PCR products were analyzed by 1% agarose gel electrophoresis and purified with the NucleoSpin^®^ Gel and PCR Clean-up kit (#740609.50, Macherey-Nagel, Germany). Afterwards, they were cloned into the pCRII vector (#K207040, TA Cloning^®^ Kit Dual Promoter with pCR^TM^II vector, Thermo Fisher Scientific, Germany), transformed into XL1-Blue competent cells (#200249, Agilent, United States), purified and sequenced with M13 primers. The obtained full length mRNA sequence of *Lm*-*crlf3* was submitted to GenBank (Accession No. MN245516) and used for gene specific primer design.

**TABLE 1:** Summary of oligonucleotides.

**Name**	**DNA sequence (5′– >3′)**
UPM long T3	5′ ATTAACCCTCACTAAAGGGAAA
	GCAGTGGTATCAACGCAGAGT 3′
UPM short T3	5′ ATTAACCCTCACTAAAGGGA 3′
RACE_*Lm-crlf3*_for	5′ GGTTCATGCTGTTGAGAGGGTTGGCAG 3′
RACE_*Lm-crlf3*_rev	5′ CTGCCAACCCTCTCAACAGCATGAACC 3′
*Lm*-crlf3 F1 Fw	5′ GTGTGATAGGTTGCCAGCAGTC 3′
*Lm*-crlf3 F1 Rv	5′ CGTATAAGGTGGTGACATTCAGGTC 3′
*Lm*-crlf3 F2 Fw	5′ GGAACCAGTCACTCTGCGAG 3′
*Lm*-*crlf3* F2 Rv	5′ CGAATATTACCCCAGGCTGGAG 3′
*Lm-rpt3 full Fw*	5′ TTGGGGATCGGTGCGTCAG 3′
*Lm-rpt3 full rV*	5′ TTATTTATAGAATTCATGCTCTGATTCATCC 3′
*Lm-rpt3* Fw	5′ GATGAGCAGCGCAATTTGAAAA 3′
*Lm-rpt3* Rv	5′ CACATCTGGCTTTTCATCTGC 3′
q*Lm-gapdh* Fw	5′ GTCTGATGACAACAGTGCAT 3′
q*Lm-gapdh* Rv	5′ GTCCATCACGCCACAACTTTC 3′
q*Lm-CRLF3* Fw	5′ GTCTGGCTCTTGCCGATCACC 3′
q*Lm-CRLF3* Rv	5′ GTAGTCTTTCCCTTGCCATCCACAAACACAC 3′
M13F	5′ GTAAAACGACGGCCAGT 3′
M13R-T7	5′ taatacgactcactataggCAGGAAACAGCTATGAC 3′

### Dissection of Locust Tissue and RNA Isolation

2–4 adult or 4 juvenile locusts were used for total RNA extraction. Brain, muscle and hemocytes RNA was isolated using the ZR Tissue & Insect RNA MicroPrep^TM^ Kit (#R2030, Zymo Research). Hemolymph (final amount 1.5 ml) was collected by injecting 500 μl anticoagulant solution (98 mM NaOH, 186 mM NaCl, 17 mM Na_2_EDTA, 41 mM citric acid, pH 4.5) into the abdomen of a locust. After 1 min, the hemolymph was collected with a pipette through an abdominal incision and stored on ice until further usage. Hemocytes were spun down, resuspended in 800 μl RNA lysis buffer and transferred to a ZR BashingBead^TM^Lysis Tube. Brains were dissected as described in Miljus et al. (2014). Skeletal muscle originated from 4 to 6 large wing muscle strands. Tissue from either brain or muscle was directly collected in 800 μl RNA lysis buffer in a ZR BashingBead^TM^Lysis Tube on ice. The following steps were performed according to the user manual including the on-column DNAse I treatment.

Total RNA from primary brain cell cultures was purified using the Monarch^®^ Total RNA Miniprep Kit (#T2010S, New England BioLabs^®^ GmbH, Germany) according to the user manual including the recommended on-column DNAse I treatment. Cells were mechanically detached from the coverslips and directly transferred into 300 μl lysis buffer. Finally, RNA was eluted twice with 20 μl nuclease free water and stored at −80°C.

### cDNA Synthesis, Reverse Transcription PCR (RT-PCR), and Quantitative Real-Time PCR (qRT-PCR)

cDNA was synthesized from 1 μg total RNA using QuantiTect Reverse Transcription Kit (#205311, Qiagen, Germany) according to the user manual. RT-PCR was performed with 100 ng cDNA template, 0.4 μM forward and reverse primers targeting *Lm-crlf3* F1 and GoTaq^®^Green Master Mix (Promega, Germany) in a 25 μl reaction volume. The PCR program consisted of an initial denaturing step at 95°C for 3 min, 30 cycles of 95°C for 30 s, 58°C for 30 s and 72°C for 45 s and a final step at 72°C for 3 min. Amplicons were analyzed by 1% agarose gel electrophoresis. qRT-PCR was conducted with the MyiQ^TM^ Single-ColorReal-Time PCRDetection System (#170-9740, Bio-Rad, Germany) in 96-well plates (#HSS9665, Bio-Rad, Germany) covered with a seal (#MSB1001, Bio-Rad, Germany). The final reaction volume was 10 μl containing 5 μl of iTaq^TM^ Universal SYBR^®^ Green Supermix (#1725121, Bio-Rad, Germany), 0.1 μM primers and 10 ng cDNA. Primers were tested for efficiency and stability. *Lm-gapdh* was used as a reference gene (Van Hiel et al., 2009). Amplification was performed with this program: 95°C for 3 min followed by 40 cycles of 95°C for 10 s, 60°C for 30 s and 72°C for 30 s, and a final step at 95°C for 1 min. Afterwards, melting curve analysis was performed starting at 55°C for 1 min and increasing the temperature in 81 cycles for 0.5°C every 10 s up to 95°C. Data were analyzed by the comparative C_T_ method (Livak and Schmittgen, 2001).

### Synthesis of Double-Stranded RNA (dsRNA)

Template DNA was amplified by PCR using M13F and M13R-T7 primers (M13R attached with an additional T7 promotor) using the following program: denaturation at 98°C for 3 min, 30 cycles of 98°C for 30 s, 60°C for 30 s and 72°C for 30 s, and a final step of 72°C for 5 min. *In vitro* transcription of dsRNA was performed using a T7 transcription kit (MEGAscript^TM^ T7 Transcription Kit, Thermo Fisher Scientific, Germany) and 400–600 ng template DNA. RNA was purified by lithium chloride precipitation and resuspended in injection buffer (1.4 mM NaCl, 0.07 mM Na_2_HPO_4_, 0.03 mM, KH_2_PO_4_, 4 mM KCl). RNA strands were annealed using a thermocycler and the following program: 60°C for 20 min, 95°C for 5 min, decrease to 20°C in steps of 0.1°C/s. Size and quality of the dsRNA was checked with 1% agarose gel electrophoresis. Prior to usage, dsRNA was filtered through a sterile filter by centrifugation (Millex^®^-HV Syringe Filter Unit, 0.45 μm, #SLHV004SL, Millipore, Germany).

### Locust Primary Brain Cell Cultures

Locust primary brain cell cultures were established from 4^th^ stage juvenile locusts as previously described (Ostrowski et al., 2011; Miljus et al., 2014). Complete growth medium consisted of L15 (Leibovitz’s L-15 Medium, #11415049, Thermo Fisher Scientific, Germany), 5% FBSG (Fetal Bovine Serum Gold, PAA Laboratories GmbH, Austria), 1x Penicillin-Streptomycin (Penicillin-Streptomycin, 10,000 units penicillin and 10 mg streptomycin/ml, #P4333, Sigma-Aldrich^®^, Germany) and 1% Amphotericin B (Gibco^TM^ Amphotericin B, 250 μg/ml, #15290018, Thermo Fisher Scientific, Germany). Dissected brains were pooled (see below), enzymatically digested with 2 mg/ml Collagenase/Dispase solution for 30–45 min at 27°C and mechanically dissociated by trituration with a 100 μl tip of an Eppendorf pipette. The primary brain cells were cultured on ConcanavalinA-coated (Sigma-Aldrich^®^, Germany) round glass cover slips (Ø 10mm, Corning, Inc., Sigma-Aldrich^®^, Germany) in 4-well NUNC plates (#176740, NuncTM Delta Surface, Thermo Fisher Scientific, Germany) filled with 500 μl of complete growth medium at 27°C in a humidified atmosphere. The medium was changed every 2 days. Based on previous studies (Gocht et al., 2009), locust brain cultures are estimated to contain approximately 3% glia and 97% neurons after 7 days *in vitro* under normoxic conditions.

### Soaking RNAi

Soaking RNAi describes the supplementation of standard growth medium with dsRNA to initiate a target-specific degradation of the respective transcripts. In order to investigate the applicability of soaking RNAi in locust primary brain cell cultures, we exposed cultures derived from the same pool of brain cells to dsRNA (final concentration 10 ng/μl) targeting various transcripts. Fresh dsRNA was added with every medium change. Cells were fixed on day 5 and stained in order to assess the effect on cell viability as described below. dsRNA targeting *Lm-rpt3* and *dsRed* were tested in this study. *Lm*-RPT3 is a proteosomal regulatory protein that is essential for cellular survival and served as a positive control for RNAi efficacy. dsRNA targeting *dsRed* served as a negative control as dsRed is not naturally expressed in *L. migratoria*. Additionally, dsRNA targeting *Lm-crlf3* fragment 1 and *Lm-crlf3* fragment 2 was applied to otherwise untreated cultures during neuroprotection assays (see below) in order to exclude effects on cellular survival of the CRLF3 knock-down itself.

### Neuroprotection Assay and Pharmacological Treatment

Neuroprotection assays compared cellular survival in cultures exposed to normoxia, hypoxia and rhEpo with or without previous knock-down of *Lm-crlf3* expression. Each experiment compared differently treated cultures that derived from the same pool of locust brain cells (two brains per culture/treatment). One experiment consisted of one culture at normoxic conditions (control), a hypoxia-treated culture (challenged, reduction to < 90% cell survival), a hypoxia- and Epo-treated culture (positive control for neuroprotective effect) and a hypoxia- and Epo-treated culture that was previously subjected to RNAi-induced *Lm-*CRLF3 knock-down (experimental group) (Figure 5). In some experiments, potential effects of dsRNA targeting *Lm-crlf3* were also determined in normoxic conditions. Control cultures were always incubated with the same medium and at the same temperature as experimental cultures. In order to knock down *Lm*-CRLF3, growth medium was supplemented with 10 ng/μl dsRNA (*Lm-crlf3* fragment 1 or fragment 2) from day 0 to day 7. After 4 days, complete growth medium was replaced by growth medium without serum. On day 5, *in vitro* cultures were treated with 32 ng/ml (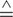 4 U/ml) rhEpo (NeoRecormon, Roche, Welwyn Garden City, United Kingdom) 12 h prior to 36 h hypoxia exposure.

Hypoxia (O_2_ level < 0.5%) was maintained in a hypoxic chamber (Hypoxia Incubator Chamber, #27310, STEMCELL,^TM^ Germany) flooded with nitrogen. After hypoxic treatment, the cells were fixed for 30 min with 4% paraformaldehyde and stained with DAPI (1:1000) without agitation as described elsewhere to assess cell viability (Miljus et al., 2014; Hahn et al., 2017). The evaluation of cell viability (at the time of fixation) was performed on the basis of the DAPI-labeled nuclear morphology (Gocht et al., 2009). Photographs were taken with a Spot CCD camera (Invisitron, Germany) mounted on an epifluorescence microscope (Zeiss Axioskop; 40x objective, Germany). Numbers of alive and dead nuclei were evaluated using Fiji (Version 1.52.i) as described elsewhere (Schindelin et al., 2012; Hahn et al., 2017). The portion of living cells was determined for each culture and normalized to the portion of living cells in the normoxic control culture (set to 1). The experimenter was blinded with respect to the identity of the cultures while cell viability evaluation.

### Statistical Analysis

Data analysis and statistics were performed with R (version 3.6.0) using R Studio (version 1.2.1335) (RStudio Team, 2018; R Core Team, 2019). Boxplots depict the median, the upper and lower quartile, and whiskers represent 1.5 times the interquartile range and outliers. Black circles represent the data of individual experiments. Statistics were calculated using the pairwise permutation test included within the packages “coin” and “rcompanion” (Hothorn et al., 2006, 2008; Mangiafico, 2019). The false discovery rate was controlled using the Benjamini–Hochberg procedure (Benjamini and Hochberg, 1995).

## Results

### Identification of *Lm-crlf3*

**FIGURE 1:**
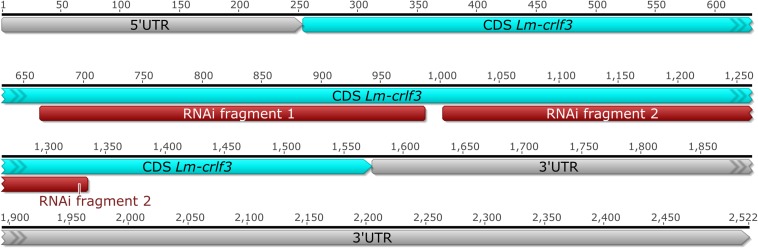
Full length brain transcript of *Lm-crlf3* obtained by RACE PCR. The coding sequence (CDS, blue) covers 1320 bp from the 2522 bp full length sequence that contains a shorter 5′ and a longer 3′ untranslated region (UTR, gray). Two non-overlapping sequences (red) were selected for RNAi-mediated knock-down *in vitro*.

The sequence of the full length *Lm-crlf3* transcript was obtained by RACE PCR from locust brain tissue. It comprises 2522 bp and includes a 253 bp 5′ UTR, a 1320 bp coding sequence (CDS) and 949 bp 3′ UTR (Figure 1). The CDS determined in Geneious^®^ refers to the translation of frame 2 and codes for 439 amino acids (see Supplementary File S1). The *Lm-crlf3* sequence was used to transcribe double-stranded RNA targeting two non-overlapping fragments for RNAi experiments, for qRT-PCR to detect *crlf3* expression in locust tissues and for phylogenetic analysis.

### *Lm-crlf3* Expression

**FIGURE 2:**
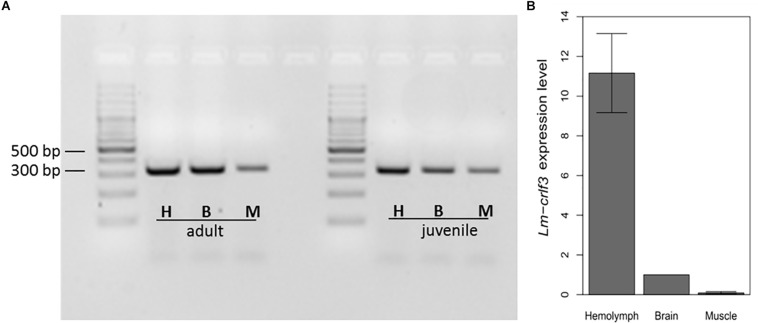
*Lm-crlf3* expression in hemocytes, brain and skeletal muscle. **(A)** 1% Agarose gel of RT-PCR products (using primers for *Lm-crlf3* fragment 1 and 100 ng cDNA template) from hemocytes (H), brain (B), and skeletal muscle (M) extracted from adult and juvenile locusts. **(B)** qRT-PCR analysis of *crlf3* expression in hemolymph, brain, and skeletal muscle of adult locusts. *Lm-gapdh* was used as a reference gene and relative expression of *Lm-crlf3* was normalized to the level of expression in the brain (set to 1). Bars display average relative expression level ± standard deviation. *N* = 3.

In mammals, *crlf3* is expressed in various tissues including the nervous system, reproductive organs, bone marrow and immune system. For comparison, *crlf3* expression was determined in brain tissue, skeletal muscle and hemolymph. As determined by RT-PCR amplifying *Lm-crlf3* fragment 1 (displayed in Figure 1), all three tissues expressed *Lm-crlf3* in detectable amounts in both adult and juvenile locusts (Figure 2A). In both developmental stages, hemolymph seemed to contain the most and muscle the least amount of *crlf3* transcripts. These semi-quantitative *crlf3* expression levels were confirmed by qRT-PCR analysis of adult locust tissues using *gapdh* as reference. With respect to brain *crlf3* expression (normalized to 1) hemolymph contained 11.16 (± 1.99 STDV) fold *crlf3* transcripts while muscle contained only 0.09 (± 0.06 STDV) fold (Figure 2B).

### Gene Tree of CRLF3

BLAST searches with the human CRLF3 query detected CRLF3 sequences with reliable e-values ranging from 0 to 6.43E-07. Coverage of the human query varied between 16% (*Pan troglodytes*) and 100%, with a median of 97.29%. The minimum sequence length was 86 amino acids (aa) (*Pan troglodytes*, Mammalia), the maximum 625 aa (*Daphnia magna*, Crustacea), whereas the median sequence length was 438 aa.

**FIGURE 3:**
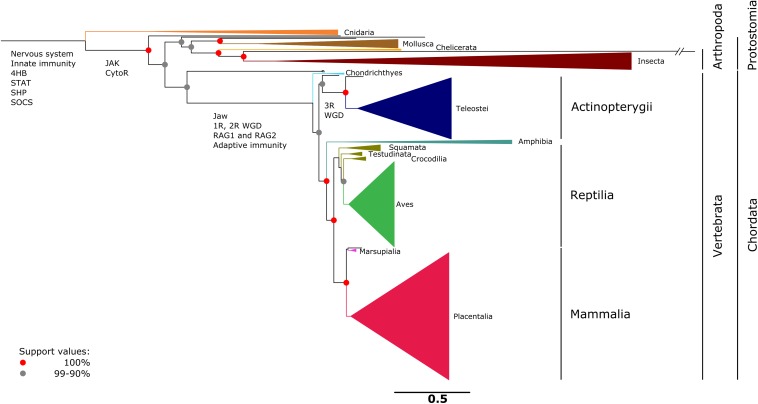
Single gene tree based on 293 amino acid sequences referring to CRLF3. CRLF3 is a well-conserved ancient protein present in eumetazoan taxa ranging from Cnidaria to Mammalia. No CRLF3 was detected in the basal metazoan taxa of Porifera, Placozoa, and Ctenophora. The topology resembles the phylogenetic relationships concluded from conventional molecular markers. For reasons of clarity, major taxa were collapsed and only the most important support values depicted as red (100%) and gray (90–99%) nodes. Important events in the evolution of the nervous system and cytokine signaling are indicated at the respective branches. A detailed tree that displays all species individually is presented as Supplementary Figure S1. The scale bar represents the expected number of amino acid substitutions per site.

CRLF3 was shown to be present in 293 eumetazoan species ranging from Cnidaria to Mammalia. No hits were found in the basal metazoan taxa Porifera, Placozoa, and Ctenophora. A collapsed version of the CRLF3-based gene tree is shown in Figure 3, while the detailed version depicting all included species is presented as Supplementary Figure S1 and Supplementary File S3. CRLF3 is present in only 34 invertebrate species while 259 hits were detected among vertebrate species. Branches are considerably longer in invertebrates than in vertebrates. Speciation events for major taxa are very well-supported (>90%).

### Soaking RNAi in Locust Primary Brain Cells

**FIGURE 4:**
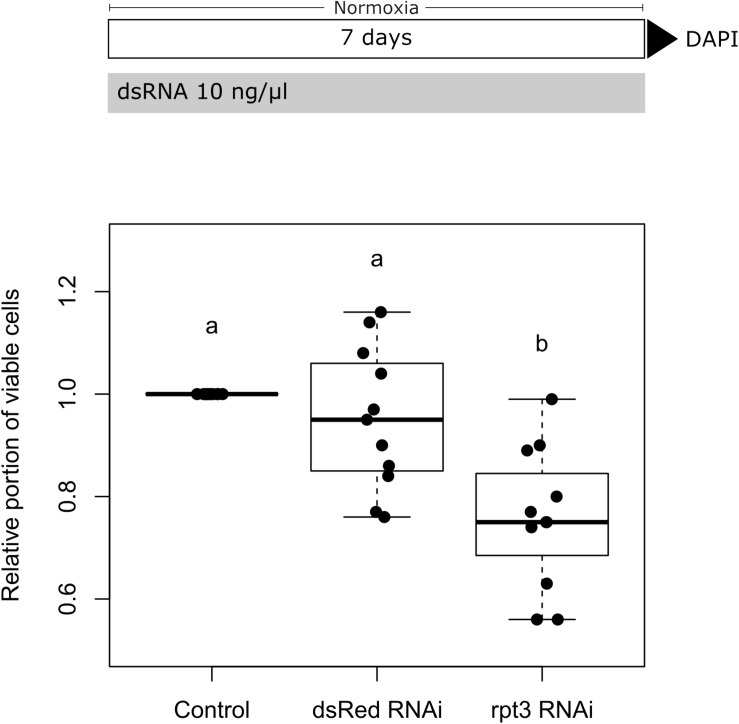
Soaking RNAi in primary brain cell cultures of *L. migratoria*. Growth medium was supplemented with 10 ng/μl dsRNA targeting either *dsRed* (not naturally present in *L. migratoria*) or *Lm-rpt3* (proteasomal protein). The growth medium was renewed every 2 days. Cell viability was assessed after 5 days *in vitro* by DAPI staining and normalized to control cultures (set to 1). Soaking cells in unspecific dsRNA (targeting *dsRed*) does not alter cell viability. Knock-down of the essential protein RPT3 significantly decreases cell viability. *N* = 11; 35,853 cells evaluated. Statistics: permutation test with Benjamini–Hochberg correction. Groups that do not share a letter (a, b) are significantly different with at least *p* < 0.05. Boxplots are complemented by data of individual experiments (black dots).

**FIGURE 5:**
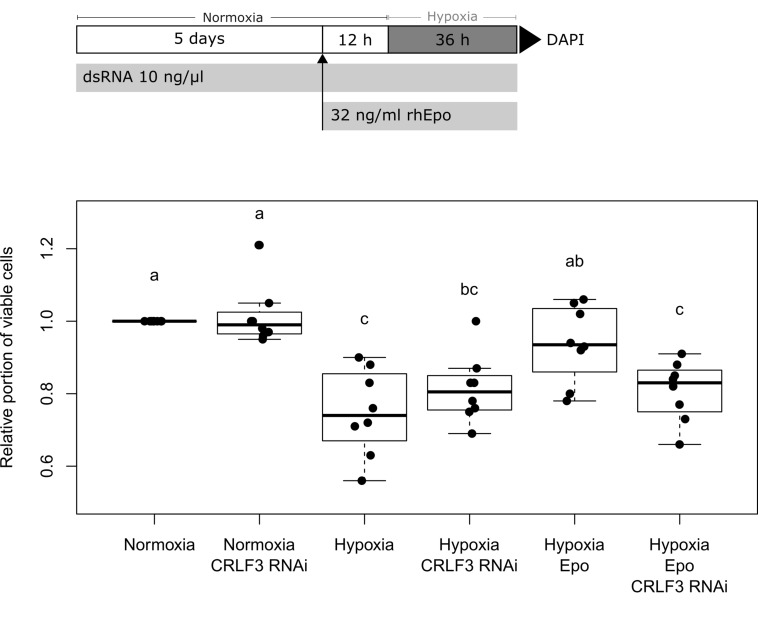
Survival of *L. migratoria* primary brain neurons in normoxia, hypoxia, and after knock-down of *Lm*-CRLF3 expression with fragment 1. Cellular survival was assessed by DAPI staining at day 7 to evaluate the impact of hypoxia (36 h), rhEpo (32 ng/ml) and RNAi-mediated knock-down of *Lm*-CRLF3 (10 ng/μl dsRNA targeting fragment 1). Hypoxia significantly decreases cell viability but treatment with rhEpo prevents neurons from hypoxia-induced apoptosis. The neuroprotective effect of rhEpo is absent following knock-down of *Lm-crlf3* expression. Knocking down *Lm*-CRLF3 *per se* has no impact on cell viability, neither in normoxia nor in hypoxia. *N* = 8; 131,792 cells evaluated. Statistics: permutation test with Benjamini–Hochberg correction. Groups that do not share a letter (a, b, c) are significantly different with at least *p* < 0.05. Boxplots are complemented by data of individual experiments (black dots).

We initially conducted control experiments to verify that cultured locust brain neurons take up dsRNA from the medium and initiate an RNAi response. We furthermore tested that soaking RNAi as such has no negative impact on cell survival. For these means, two dsRNA constructs (applied at 10 ng/μl concentration for 5 days) were evaluated in respect to their effect on neuronal cell survival. The first one targeted dsRed, a protein that is not naturally expressed in *L. migratoria*. As shown in Figure 4, dsRNA targeting dsRed had no significant effect on cell survival. In contrast, dsRNA targeting the expression of the proteasomal protein rpt3 caused a significant reduction of cellular survival (*p* = 0.0008; median survival 74.8% compared to untreated controls from the same pool of cells). In addition, dsRNA targeting CRLF3 had no significant effect on cellular survival compared to untreated control cultures in both unchallenged and challenged cell cultures (Figure 5 and Supplementary Figure S3). These results indicate the functionality of soaking RNAi to suppress the translation of target proteins and serve as a control for the negligible impact of unspecific dsRNA and its solvent on cell viability.

### Involvement of *Lm-*CRLF3 in Epo-Mediated Neuroprotection

After demonstrating a robust knock-down of *Lm*-*rpt3* following 5 days of exposure to dsRNA, we knocked down *Lm-crlf3* expression to investigate its importance for Epo-mediated neuroprotection. In these experiments, we applied dsRNA with the same protocol (exposure to 10 ng/μl dsRNA for 5 days before experiments started) targeting two non-overlapping fragments of *Lm-crlf3* to exclude off-target effects. qRT-PCR analysis proved that after 5 days of soaking RNAi the expression levels dropped by half compared to controls (Supplementary Figure S2). The involvement of CRLF3 in Epo-mediated neuroprotection of locust neurons was tested in a neuroprotection assay. Following identical protocols, two series of experiments were conducted, in which *Lm*-CRLF3 expression was suppressed by two different dsRNA constructs (fragment 1 and fragment 2). The results of experiments with fragment 1 are shown in Figure 5 whereas the results of experiments with fragment 2 are displayed in Supplementary Figure S3. Cells were stressed by hypoxia (<0.5% O_2_) and a normoxic group was used as control (cell viability was set to 1). Cell viability was significantly decreased by hypoxia, however, pre-treatment with 32 ng/μl rhEpo protected cells from hypoxia-induced apoptosis. Knock-down of CRLF3 abolished Epo’s neuroprotective effect and cell viability was not significantly different to sole hypoxia-treated cells. The phenotypes observed by soaking RNAi using dsRNA constructs targeting *Lm-crlf3* fragment 1 or fragment 2 are similar (compare Figure 5 and Supplementary Figure S3). Hence, unspecific off-target effects resulting from interference with the expression of another protein are unlikely.

## Discussion

*Lm*-CRLF3 consists of 439 amino acids (aa) which is similar to the size of other insect CRLF3 sequences ranging from 391 to 504 aa. The size of all CRLF3 sequences included into the analysis varies between 86 and 625 aa. CRLF3 found in *Gryllus bimaculatus*, another orthopteran species, is the closest to *Lm*-CRLF3 (51% identity). Similar to *Lm*-CRLF3, the *T. castaneum* CRLF3 receptor (*Tc*-CRLF3) has previously been described as an Epo-responsive receptor involved in neuroprotection (Hahn et al., 2017). Both receptors share 35% similarity of their sequences while the similarity between the locust and the human sequence is 29%.

### Phylogenetic Analysis of CRLF3 and Potential Functions

CRLF3 sequences were identified in 293 species and included in the phylogenetic analysis. The single gene tree mirrors the phylogenetic relationships of major metazoan taxa concluded from studies using conventional molecular markers or transcriptomes with remarkable detail (Dunn et al., 2014; Misof et al., 2014; Irie et al., 2018; Laumer et al., 2019). However, minor exceptions are present. The chordate *Ciona intestinalis* is represented as a sister group to all bilaterians but has been shown to be the sister group to vertebrates (Berná et al., 2009). The misplacement of *Ciona* might be explained by its particularly fast evolving genome (Berná et al., 2009). Furthermore, *Latimeria chalumnae* appears as sister to ray-finned fish (Actinopterygii). For decades, the phylogenetic position of *Latimeria* was subject to debate, but recent studies provide evidence for a closer relationship of *Latimeria* to tetrapods (Takezaki and Nishihara, 2016; Yoshida et al., 2019).

The high conservation of CRLF3 in vertebrates, reflected by short branches, suggests an important role for the organisms. Since the phylogenetic tree of CRLF3 resembles the molecular metazoan tree of life, CRLF3 seems to be subjected to rather high selective pressure leading to a fairly low evolutionary rate. The fact that no matches were found in Porifera, Placozoa and Ctenophora but in Cnidaria, suggests that CRLF3 might have evolved for some function in eumetazoan nervous systems (Grimmelikhuijzen et al., 1996; Westfall, 1996; Bosch et al., 2017). Porifera and Placozoa lack nervous systems. Nervous systems of Ctenophora are fundamentally different from eumetazoan counterparts (with respect to the presence of typical transmitters, gap-junctional proteins, expression of *elav*, large number of specific neuropeptides and others) (Moroz and Kohn, 2016). Thus, an independent evolutionary origin of ctenophoran and eumetazoan nervous systems is intensely debated (Jékely et al., 2015; Moroz and Kohn, 2016). Cnidaria possess extended neuronal networks that are regarded as homologous to the bilaterian nervous system. The presence of CRLF3 in Eumetazoa but not in Ctenophora matches the hypothesis that the nervous systems of both taxa have evolved convergently (Jékely et al., 2015; Moroz and Kohn, 2016). Main components of the CRLF3-initiated signaling cascades were already present in ancestors of Cnidarians, including the four-helix-bundle (characteristic of class-I cytokines), SHP, STAT, and SOCS. These might have been complemented by JAK and CytoR in Bilateria forming the JAK/STAT signaling pathway (Babonis and Martindale, 2016; Liongue et al., 2016).

Although many invertebrate genomes (626 in NCBI) are available, CRLF3 is only sparsely present. Furthermore, the branch lengths amongst invertebrate taxa are comparably long indicating a lower conservation and higher diversification of this gene (Supplementary Figure S1). In order to exclude that the sparse representation of CRLF3 in insects is due to sequence unavailability and poor quality, we performed an additional analysis restricted to insects (data not shown). We analyzed the transcriptomes published by Misof and colleagues, who provided a robust phylogenetic tree based on high quality transcriptomes covering all extant insect orders and some other arthropods (144 in total) (Misof et al., 2014). Evaluation of this data set confirmed the low abundance of CRLF3 in insects (5 species out of 128). Hence, we hypothesize that CRLF3 has been lost in many invertebrates but was strictly maintained in vertebrates. Potential involvement in further physiological processes outside the nervous system, for instance within adaptive immunity, might have increased the selective pressure. In contrast to the innate immune system, which has already been present in early eukaryotes, the adaptive immune system, relying on V(D)J recombination, emerged in jawed vertebrates 450 million years ago (Rast et al., 1997; Dzik, 2010). It has been suggested that this adaptive immune system is an offshoot of the nervous system or that both derived from an ancestral neuro-immune cell (Bayne, 2003; Kioussis and Pachnis, 2009).

Both, the nervous and adaptive immune system share a variety of signaling molecules including neurotrophic factors, cytokines, chemokines (Habibi et al., 2009; Kerschensteiner et al., 2009; Ransohoff, 2009) and their receptors (Atwal et al., 2008; Levite, 2008; Ben Baruch-Morgenstern et al., 2014). Some molecules have initially been assigned as neurospecific and were later found to be involved in immune functions. For instance, the neuropeptide Y (orthologs are already present in insects) was first discovered as one of the most abundant neuropeptides in the central nervous system but has additional effects on immune cells (Brown et al., 1999; Wheway et al., 2007). In addition, the proteoglycan agrin was known to be required for the formation of the neuromuscular junction but is present on lymphocytes, too (Gautam et al., 1996; Khan et al., 2001; Zhang et al., 2006). In contrast, interleukin 2 was introduced as an immunoregulatory cytokine but more recent studies detected interleukin 2 production by neurons (Muraguchi et al., 1985; Meola et al., 2013). These shared chemical molecules involved in cell-to-cell communication support the hypothesis of an evolutionary common origin of the nervous and adaptive immune system.

Besides functional and physiological similarities, they also share morphological similarities. Both systems function via intimate associations, called synapses, at interfaces between homologous and heterologous cells. The term immunological synapse refers to the similarity to neural synapses and describes the contact between a T cell and an antigen presenting cell. The neural synapse and the immunological synapse share structural (e.g., adhesion molecules, cytokine secretion, receptor clustering) and functional (e.g., memory storage, exchange of information) commonalities (Donnadieu et al., 2001; Habibi et al., 2009). For instance, the proteoglycan agrin plays an important role in the construction and regulation of synapse formation in both synapse types (Gautam et al., 1996; Khan et al., 2001).

A major trigger for the development of this offshoot could have been two rounds of whole genome duplications (WGDs) that occurred after the split of invertebrates and the evolution toward jawed vertebrates (Gnathostomata) (Figure 3 and Supplementary Figure S1). According to the 2R hypothesis, the first WGD took place within early chordates and the second in the last common ancestor of gnathosthomes (Kasahara, 2007). WGDs facilitate the adaptation of genes and molecules to new functions and cooption into new tasks. After WGD, one copy becomes redundant lowering the selection pressure on the maintenance of its original function. This copy might either accumulate adverse mutations or gain beneficial modifications that enable the acquisition of new functions.

Furthermore, a horizontal gene transfer of a bacterial transposon after the second WGD might have led to the incorporation of the recombination activating genes (RAGs) *RAG1* and *RAG2* providing the basis for somatic V(D)J recombination within the adaptive immune system (Oettinger et al., 1990; Agrawal et al., 1998; Schatz, 2004; Kapitonov and Koonin, 2015). The V(D)J recombination occurs only in lymphocyte development generating the diverse repertoire of antigen receptors that are required for adaptive immunity (Oettinger et al., 1990). In this context, the orphan cytokine receptor might have been adapted to new functions of the adaptive immune system of jawed vertebrates. This hypothesis is supported by data indicating that WGDs diversified the functions of Tyrosine receptor kinases (Brunet et al., 2016). Furthermore, other class I cytokine receptors besides CRLF3 are also involved in the immune system (Holdsworth and Gan, 2015). The lack of this adaptive immunity in invertebrates might have altered the selective pressure on CRLF3. This hypothesis would explain the sparse occurrence as well as the high diversity seen in these species.

### *Lm-crlf3* Is Expressed in Various Tissues

In mammals, CRLF3 is expressed in a variety of tissues including the kidney, pancreas, and brain (Yang et al., 2009). Cell protective functions of Epo have also been reported in various tissues including the hematopoietic system, kidney, skeletal and heart muscle, pancreas, and others all of which also express CRLF3 (Brines and Cerami, 2006; Noguchi et al., 2008; Yang et al., 2009; Ogunshola and Bogdanova, 2013). This supports the hypothesis that CRLF3 might be a cell protective Epo receptor in mammals. In addition, similar expression patterns have been detected in various locust tissues including the nervous system, hemolymph, and skeletal muscle. Since hemocytes display adhesive properties, it cannot be excluded that low levels of *crlf3* transcripts detected in wing muscle may result from contamination with adherent hemocytes that circulate throughout the hemocoel. This data suggests that CRLF3 is an ancient receptor that in the beginning had a function in general cell protective mechanisms and has been adapted to various tissues during evolution.

### Soaking RNAi for Loss of Function Studies in Locust Neurons

We supplemented cell culture medium with dsRNA and let locust primary brain cells spontaneously take up dsRNA for 5 days. Medium and dsRNA were refreshed every 2 days to maintain a constant supply of dsRNA and nutrients. In order to assess the applicability of soaking RNAi in locust neuron cultures, we targeted the proteasomal protein RPT3 that is essential for cellular survival. The reduction of cellular survival to 75%, compared to untreated control cultures, indicates that the neurons take up dsRNA and process it to small interfering RNA (siRNA) initiating mRNA degradation. dsRNA targeting dsRed, a protein that is not expressed in locusts, showed no significant reduction in cellular survival. By this we show that cell death was caused by the absence of RPT3 and not due to effects that were associated with dsRNA uptake. Moreover, dsRNA-mediated knock-down of *Lm-crlf3* expression had no significant impact on neuronal survival, neither in normoxic nor hypoxic conditions. These results indicate that changes in cellular survival depended on the absence of specifically downregulated proteins [RPT3 and CRLF3 during hypoxia/Epo treatment (see below)] rather than on unspecific effects of dsRNA exposure.

RNAi in insects has been reported by various studies (reviewed in Vogel et al., 2019). Many of them focused on RNAi as a tool for pest control (Mamta and Rajam, 2017; Niu et al., 2018). However, the efficiency and success of RNAi varies tremendously between species and targeted tissues. Presumably, this depends on differences in dsRNA uptake and the ability to process dsRNA to siRNA (Ren et al., 2014; Wang et al., 2016; Singh et al., 2017). *In vivo*, dsRNA is typically delivered by feeding or injection into the hemocoel. It has been shown that dsRNAses are more abundant in the digestive system than in hemolymph. This is in line with observations showing that feeding dsRNA is often less effective than injecting dsRNA (Wang et al., 2016; Singh et al., 2017; Song et al., 2019). In locusts, feeding of dsRNA is not successful whereas injecting dsRNA leads to a robust systemic RNAi response (Luo et al., 2013; Song et al., 2019; Xie et al., 2019). The efficacy of RNAi differs between tissues. Injection of dsRNA into the hemocoel induces RNAi in the brain but not in ovaries (Ma et al., 2011; Ren et al., 2014). We herein introduce a new and convenient RNAi application method for loss of function studies in locust primary brain cell cultures. We termed it soaking RNAi since it requires no additional manipulations (e.g., lipofection, electroporation, viral delivery) to suppress specific protein expression as in mammalian cells. Soaking RNAi has been successfully applied in primary brain cell cultures from the beetle *T. castaneum* (Hahn et al., 2017). It has now been adapted to locust primary brain neurons, offering a new tool for *in vitro* loss of function studies in *L. migratoria*. The RNAi effect observed in *L. migratoria* is slightly lower than in the beetle *T. castaneum* (Hahn et al., 2017). Four days exposure of *T. castaneum* brain cells to 10 ng/μl dsRNA targeting *rpt3* expression reduced cell survival to approximately 34–67% while 5 days exposure reduced *L. migratoria* median brain cell survival to approximately 75%. In line with these observations, coleoptera, including *T. castaneum*, exhibit a generally higher RNAi susceptibility in comparison to *L. migratoria* (Wang et al., 2016; Singh et al., 2017). However, the RNAi response of *L. migratoria* is sufficiently robust and useful for loss of function studies.

### *Lm*-CRLF3 Is Crucial for Epo-Mediated Neuroprotection

Selective neuroprotective activity (without stimulation of erythropoiesis) by EV-3 and other Epo-like ligands (e.g., carbamylated Epo, asialo-Epo, helix B surface peptide, Epo mimetic peptide 1) provided clear evidence for alternative cell protective Epo-receptors other than (EpoR)_2_ (Erbayraktar et al., 2003; Brines et al., 2004, 2008; Leist et al., 2004; Bonnas et al., 2017). Several receptors and receptor complexes have been associated with Epo-induced neuroprotection in mammals, including homodimeric (EpoR)_2_, heteromeric EpoR/β-common receptor and Ephrin B4 receptor (Brines et al., 2004; Um et al., 2007; Pradeep et al., 2016). However, Epo-mediated neuroprotection remains only partially understood. Hence, we investigated a potential neuroprotective involvement of CRLF3 in locust primary brain neurons.

Our experiments indicate that *Lm*-CRLF3 represents an Epo-binding receptor, or alternatively constitutes an essential component of an Epo-binding receptor complex, whose activation can fully prevent hypoxia-induced apoptosis in locust primary brain cell cultures. RNAi against *Lm-crlf3* does not generally affect the cell viability of locust primary brain neurons, neither in unchallenged nor in challenged conditions. This implies that *Lm-*CRLF3 is not involved in physiological maintenance of differentiated neurons, but specifically induces protective mechanisms upon Epo stimulation. However, *Lm*-CRLF3 is crucial for Epo-induced neuroprotection *in vitro* since a knock-down by RNAi abolished the neuroprotective effect of Epo in locust primary brain cell cultures. Our previous study focused on the holometabolous beetle *T. castaneum* and saw similar results concerning CRLF3 involvement. Given that locusts are hemimetabolous, these findings lead to the assumption that the last common ancestor of hemi- and holometabolous insects already employed CRLF3 as a neuroprotective receptor. However, its endogenous ligand is yet unknown. Since insects do not possess *epo* genes, the endogenous ligand has to be different from Epo but might share structural features with Epo and other class-I helical cytokines. The artificial activation of insect CRLF3 receptors by rhEpo is not surprising, because CRLF3 and EpoR both belong to group 1 of class I cytokine receptors and EpoR has already been shown to crossreact with thrombopoetin, the typical ligand of another receptor of that group (Rouleau et al., 2004).

In contrast to vertebrates, only few cytokines or cytokine-like peptides have been identified in insects (Duressa et al., 2015; Schrag et al., 2017; Matsumura et al., 2018). Expression of CRLF3 by locust hemocytes and brain cells may indicate multiple production sites of its endogenous ligand, since blood-brain-barriers restrict the exchange of ions and soluble molecules (reviewed in DeSalvo et al., 2011). Potential production sites, that release the ligand into the circulation, are certain types of hemocytes, neurosecretory organs including the corpora allata as well as the corpora cardiaca, and the fat body. They contain and release also other cytokines involved in stress responses (Duressa et al., 2015; Matsumura et al., 2018).

As documented for many species from different orders, insects achieve extraordinary resistance to hypoxia by switching to anaerobic metabolic pathways and reduction of basal metabolic rates amongst other adaptations (reviewed by Hoback and Stanley, 2001). Hypoxia tolerance has also been reported for locusts (Arieli and Lehrer, 1988; Wegener and Moratzky, 1995; Greenlee and Harrison, 2004) and *T. castaneum* (Donahaye, 1990; Kharel et al., 2019), the two species in which CRLF3-mediated neuroprotection has been demonstrated. In comparison to mammalian neurons, where rather brief hypoxic episodes are sufficient to induce apoptosis, survival of locust and beetle neurons *in vitro* decreases only 20–30% even when challenged by prolonged (36 h) and severe (<0.3% oxygen) hypoxia (this study; Miljus et al., 2014; Hahn et al., 2017). However, similar degrees of hypoxia tolerance have also been reported for specially adapted vertebrates (such as turtles and naked mole-rats; reviewed by Larson et al., 2014) and for some mammalian cell types *in vitro* (human SH-SY5Y neuroblastoma cells, Reich et al., 2008). Since DAPI nuclear staining, trypan blue accumulation and immunocytochemical detection of pro-apoptotic activated caspase-3 consistently identified dead or dying locust neurons (Gocht et al., 2009; Miljus et al., 2014; Heinrich et al., 2017), hypoxia-induced cell death is most likely not underestimated by our analysis of DAPI-labeled nuclear morphology. Whether and how CRLF3-induced adaptations may contribute to hypoxia tolerance *in vivo* will be a subject of our future studies.

The neuroprotective effect of rhEpo on locust neurons challenged by hypoxia, by the cellular toxin H-7 or by serum deprivation has been characterized earlier. Its anti-apoptotic mechanisms involve activation of JAK/STAT signaling, translation of anti-apoptotic factors and interference with caspase-activation but are independent of PI3K signaling (Miljus et al., 2014; Heinrich et al., 2017). At that time, the receptor mediating this effect was not known. It can be assumed, that these insights are transferable to neuroprotective CRLF3 signaling since the knock-down of CRLF3 in beetles and locusts brain cell cultures abolished Epo-mediated neuroprotection completely, suggesting that CRLF3 is the only neuroprotective Epo-receptor in these insect neurons. Furthermore, it is likely that even the neuroprotective but non-erythropoietic Epo variants (e.g., EV-3) and Epo like ligands activate CRLF3. Epo-induced endocytosis is reduced by pre-incubation to EV-3 indicating that EV-3 and Epo bind to the same receptor on locust neurons (Miljus et al., 2017).

Its sensitivity toward Epo treatment in insects and its high conservation throughout metazoans suggests CRLF3 as a potential mammalian neuroprotective Epo-receptor. The neuroprotective function of Epo has been well-investigated in vertebrates and Epo is even used in clinical trials as a treatment after ischemic stroke (Ehrenreich et al., 2009; Subiras et al., 2012; Habib et al., 2019; Simon et al., 2019). However, Epo treatment leads to various adverse side effects (e.g., thromboembolism, cardiovascular events) that mainly arise from its erythropoietic function in vertebrates (Jelkmann et al., 2008; Noguchi et al., 2008; Ehrenreich et al., 2009; Souvenir et al., 2015). Hence, developing drugs that specifically target CRLF3 might improve neuroprotective therapies.

### Outlook

Current experiments focus on the identification of the endogenous ligand of CRLF3 and the characterization of mammalian CRLF3. Cytokines typically share low sequence and overall structural similarity which complicates analyses of their evolutionary origins (Beschin et al., 2001; Liongue and Ward, 2007). Instead of bioinformatic approaches based on sequence similarity, endogenous CRLF3 ligands may rather be identified by functional studies with fractionated tissue extracts from which potential ligands can be separated and molecularly identified (Watari et al., 2019). In addition, potential neuro- and cell protective functions of CRLF3 in Mammalia should be investigated also considering a putative involvement in the adaptive immune system.

## Data Availability Statement

Nucleotide sequences were submitted to GenBank with the submission numbers MN245516 and MN245517.

## Author Contributions

NH, LB, NS-D, BM, PN, and RH collected the experimental data. NH, LB, BG, and RH conducted the data analysis and interpretation. NH and SB performed the phylogenetic analysis. NH, MG, and RH wrote the manuscript. NH and RH designed and supervised the study. All authors discussed the results and commented on the manuscript.

## Conflict of Interest

RH is a consultant for Epomedics GmbH, Göttingen, Germany. The remaining authors declare that the research was conducted in the absence of any commercial or financial relationships that could be construed as a potential conflict of interest.

## Footnotes

1 http://n2t.net/addgene:68202

2 http://www.locustmine.org/viroblast/viroblast.php

3 http://asgard.rc.fas.harvard.edu/blast.html

4 https://i5k.nal.usda.gov/locusta-migratoria
