# Supplementary figures and images for: The Orphan Cytokine Receptor CRLF3 Emerged With the Origin of the Nervous System and Is a Neuroprotective Erythropoietin Receptor in Locusts
Source: Front Mol Neurosci. 2019 Oct 11;12:251. doi: 10.3389/fnmol.2019.00251 (PMC6797617; doi:10.3389/fnmol.2019.00251)

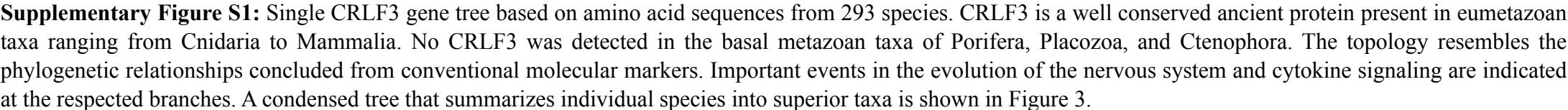

Supplement: Supplementary file 1 [file Data_Sheet_1.PDF]
