# Supplementary material for: The Orphan Cytokine Receptor CRLF3 Emerged With the Origin of the Nervous System and Is a Neuroprotective Erythropoietin Receptor in Locusts
Source: Front Mol Neurosci. 2019 Oct 11;12:251. doi: 10.3389/fnmol.2019.00251 (PMC6797617; doi:10.3389/fnmol.2019.00251)
Supplement: Supplementary file 2 [file Data_Sheet_2.PDF]

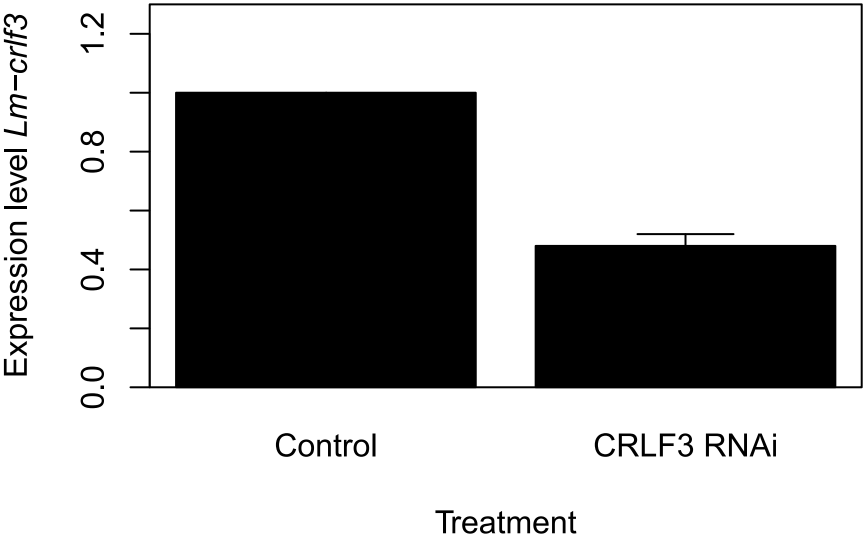

Supplementary Figure S2: Verification of *Lm-crlf3* knock-down by qRT-PCR after 5 days of soaking RNAi. Cells were incubated with injection buffer (control) or dsRNA targeting *Lm-crlf3* fragment 1. Mean = 0.48, standard deviation = 0.04, N = 2.
