# Supplementary material for: The Orphan Cytokine Receptor CRLF3 Emerged With the Origin of the Nervous System and Is a Neuroprotective Erythropoietin Receptor in Locusts
Source: Front Mol Neurosci. 2019 Oct 11;12:251. doi: 10.3389/fnmol.2019.00251 (PMC6797617; doi:10.3389/fnmol.2019.00251)
Supplement: Supplementary file 3 [file Data_Sheet_3.PDF]

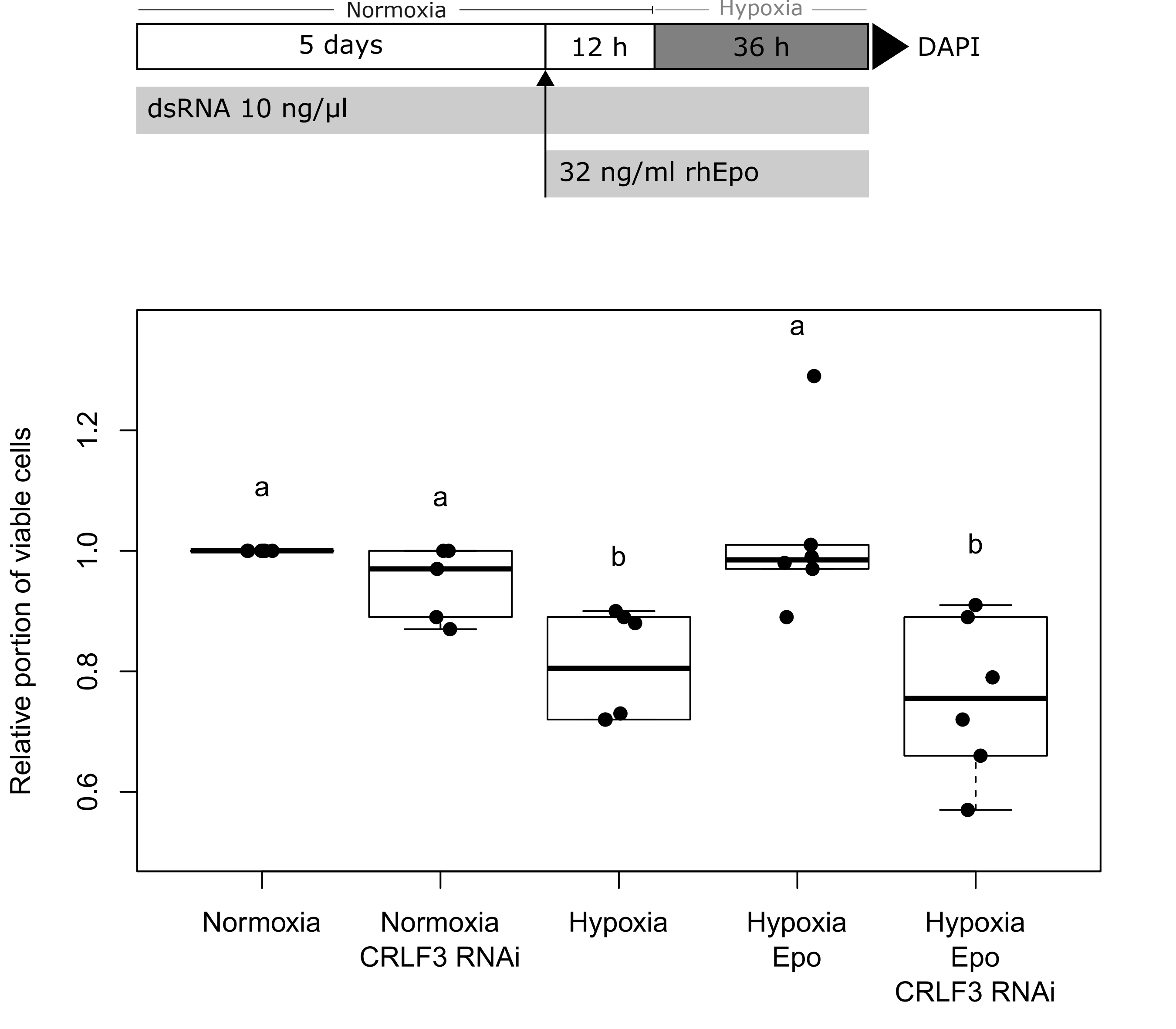

Supplementary Figure S3: Survival of *L. migratoria* primary brain neurons in normoxia, hypoxia and after knock-down of *Lm*-CRLF3 expression with fragment 2. Cell survival was assessed by DAPI staining on day 7 to evaluate the impact of hypoxia (36 h), rhEpo (32 ng/ml) and RNAi-mediated knock-down of *Lm*-CRLF3 production (10 ng/µl dsRNA targeting fragment 2). Hypoxia significantly decreases cell viability but treatment with rhEpo prevents neurons from hypoxia-induced apoptosis. The neuroprotective effect of rhEpo is absent following knock-down of *Lm*-CRLF3 expression. Knocking down *Lm*-CRLF3 *per se* has no impact on cell viability in normoxia. N= 6 except normoxia RNAi N=5; 28,216 cells counted. Statistics: Permutation test with Benjamini-Hochberg correction. Groups that do not share a letter (a, b) are significantly different with at least $p<0.05$. Boxplots are complemented by black dots that represent individual experiments.
