# Supplementary material for: The Orphan Cytokine Receptor CRLF3 Emerged With the Origin of the Nervous System and Is a Neuroprotective Erythropoietin Receptor in Locusts
Source: Front Mol Neurosci. 2019 Oct 11;12:251. doi: 10.3389/fnmol.2019.00251 (PMC6797617; doi:10.3389/fnmol.2019.00251)
Supplement: Supplementary file 4 [file Table_1.DOCX]

## Full-length mRNA *Lm-crlf3* (Accession Number MN245516)

ACATGGGGGTAATCCTGATGTTTTCTGAGGGACATTTGAACTAGTAGGAAAATAAGCGGATTGTTTAAGTTTGCTCTAAGTATTCTTCTAAAGGAGGATATGAAAAGATTTATGAGCTCTGAAAAGTCTACTATGTGGTTGAAACGATATTTTTCCCGCTCCTGTAGTGTGCCGGAAATTAAGATTTTCTGAAGGCGTGGCAGATTTTCATTAAATTTTTAAATATTCGTTTAATTTGCTTTCTTTGATTGAAATGGAACAGTCAAAGTCAAATAGTGTTTTTGTCGAAGCTGTCGATGCTGCTGAGGAATATGTGAAAAAATTGGAAGATCTACTGAAAACCCTTACTGAAGCAGAACATCAAGTGAAGCACAGTGCCAAGCAGGCAAGAGCAGATGTCAATTTTGCATTGGACACACTAGCAGCAACTTTCACTAATGCTTTAAAGCGAAGGCGAAGCATGCTCCTTGAAGAAGTGGATCGTTTGTGTAGTGAAGGACTGGAACCTCTAATTGAATGTAGAGATCTTATTACTACTCAAATGCAAGTGGCCCACAGCTATGCTAATGAAGGGCGGCATTCTCTAAATGGCAGTAACAAATGTCCAGTTGCTGAAAGCTGGGATGACTACACTCACTCATATGCAGAGTGTGGAGCTGCATTTCTTGGAAGGTTGCCAGCAGTCCCAAATTTGGAAGATATTCCTGGTGTGCAGTTTGAATGCTGTCTACATGCCATTGAAGCTGACTTGGTTCATGCTGTTGAGAGGGTTGGCAGTGTGTCACGACTTGGACCTGTCCAAATTTGTGCTGTTGAAGAGAAACCTGGAGCTCTGCTGGTCCATTGGCAGCAGGTAGAGACAGAGAAACCAGCAGAAATTGGCTCATTCAGGCTACAACGAGCATACGGAGATGCACGTGGCCGTCGTGAGTTAGAAGCCAATTTCCACGATGAGTATGTGGGACCTGAATGTCACCACCTTATACGCAATCTGCGACCAAAGGAACCAGTCACTCTGCGAGTCTGTTGCCGTGAAGACAGTGAGCTTGCACCGTGGAGTACTTGGTCTGTGTTACATGTTGCTGCAACCTCACTACCTCCATTCTGCTGGGAGGGAACAAATCAGAACTACACTATAACAAATGAGAAACAACTGGCCACAAAAAGCACCGCAGAAACGTCTGTCCTTTTTTCATGTGGGCCACAATTTGGACCAGGACATGCAGTGGAATTCACTGTTCTAGAATGTGGTGCAGGATGTAGTGACGAAGGTCTGGCTCTTGCCGATCACCATTTTAGTGATGAAAATCTTCTCCAGCCTGGGGTAATATTCGTTAATGCACAAGGAAGTGTGTTTGTGGATGGCAAGGGAAAGACTACCAAATTACCTCCCTTAGAAAAAGGCTCAAAACTATGTTTCACTTGTGAACATGTCCGCAGTAGTAAGGTCCGTATCCACATTGACAGTGGTAATAAGACTGTAGCGTATGACTGGAATGTTAGTAGTCCACTGCAAAAACTATTTTTTGCTATCTCTTTTGGACAAGTTGGCTGGAAGGTGTTGGTTGAATAAGAAAAATGGTAATTACAGATTACTTCACAAAAAAATTAAGGAATTTATATTTTGCTACAATTATACTTTTATACTGTAGCAGCCTTGAAGGTACCAMCAAGCAGTCTCATAATTTTTGTTGTTCATCCCATCGATACTATACTACTTCCAATGTGTTCAGTTTTCAGTACGTGGATATTTCATTCGTTGGCTGTAATTTTTAAATAGTGTCAGTTATCCAATTAGGTAACCTTCCAAAAAGCAGTTATCCATTGGGATAATTTATTTTGTGTGTGTGTGTGTGTGTGTGTGTGTGTGTGTGTGTTTGTTTTTTAATTTTGTGTTTGAGTGTTATTATTTGAATYTGAATACCAATTTGTTTTTGTATCACTCTATTTTCATTTGGTGGTTTTTTTCCTGATGCTTAAACGTTTAGCAGAATTAGCATAATAGTTTTTTYTTGATGGTCAATGCTTTATTGYTATGATAGAATWTAATATACCCCTAATTGCTTACATACCTGKGAAWATACATTATATWTCTTACCCTTTTGTTTGTTMCCCCTTTTTAWAATTATGTTAGKGKGATTAAGGTAAAACTTCMCATTTCYTAATCMCTAAATWTTTCCAAGTSCCTTGCTTTAASCMCTYCMCTGTGTTGTTGTTAATTTTTTTTMCCTAAATGKGKGAAGKGTTTGTMCAACCAATTCATGTAGKGTYTAAAGGACAAYTAGKGCATAATTTWTTGAMCATTYTATACTGCTTTYTTGACAMCATTGGKGTTCWTYTCYTYTGAACAAATTGTACATAGGKGAMCMCTTAAATTWTTTAMCAGATGTTTTTTYTGRGAAATGTTATATATAMCAATTMCAATATATATATAAAATTWTTAAAAAATTGTTATGCATTTTGMCAAAAWAAAGAATTTTTAAAATAAAAAAAAAAAAAAAAAAAAAAAAAAAAAAAAAAGT

## CDS Lm-crlf3

TTGCTTTCTTTGATTGAAATGGAACAGTCAAAGTCAAATAGTGTTTTTGTCGAAGCTGTCGATGCTGCTGAGGAATATGTGAAAAAATTGGAAGATCTACTGAAAACCCTTACTGAAGCAGAACATCAAGTGAAGCACAGTGCCAAGCAGGCAAGAGCAGATGTCAATTTTGCATTGGACACACTAGCAGCAACTTTCACTAATGCTTTAAAGCGAAGGCGAAGCATGCTCCTTGAAGAAGTGGATCGTTTGTGTAGTGAAGGACTGGAACCTCTAATTGAATGTAGAGATCTTATTACTACTCAAATGCAAGTGGCCCACAGCTATGCTAATGAAGGGCGGCATTCTCTAAATGGCAGTAACAAATGTCCAGTTGCTGAAAGCTGGGATGACTACACTCACTCATATGCAGAGTGTGGAGCTGCATTTCTTGGAAGGTTGCCAGCAGTCCCAAATTTGGAAGATATTCCTGGTGTGCAGTTTGAATGCTGTCTACATGCCATTGAAGCTGACTTGGTTCATGCTGTTGAGAGGGTTGGCAGTGTGTCACGACTTGGACCTGTCCAAATTTGTGCTGTTGAAGAGAAACCTGGAGCTCTGCTGGTCCATTGGCAGCAGGTAGAGACAGAGAAACCAGCAGAAATTGGCTCATTCAGGCTACAACGAGCATACGGAGATGCACGTGGCCGTCGTGAGTTAGAAGCCAATTTCCACGATGAGTATGTGGGACCTGAATGTCACCACCTTATACGCAATCTGCGACCAAAGGAACCAGTCACTCTGCGAGTCTGTTGCCGTGAAGACAGTGAGCTTGCACCGTGGAGTACTTGGTCTGTGTTACATGTTGCTGCAACCTCACTACCTCCATTCTGCTGGGAGGGAACAAATCAGAACTACACTATAACAAATGAGAAACAACTGGCCACAAAAAGCACCGCAGAAACGTCTGTCCTTTTTTCATGTGGGCCACAATTTGGACCAGGACATGCAGTGGAATTCACTGTTCTAGAATGTGGTGCAGGATGTAGTGACGAAGGTCTGGCTCTTGCCGATCACCATTTTAGTGATGAAAATCTTCTCCAGCCTGGGGTAATATTCGTTAATGCACAAGGAAGTGTGTTTGTGGATGGCAAGGGAAAGACTACCAAATTACCTCCCTTAGAAAAAGGCTCAAAACTATGTTTCACTTGTGAACATGTCCGCAGTAGTAAGGTCCGTATCCACATTGACAGTGGTAATAAGACTGTAGCGTATGACTGGAATGTTAGTAGTCCACTGCAAAAACTATTTTTTGCTATCTCTTTTGGACAAGTTGGCTGGAAGGTGTTGGTTGAATAA

## RNAi *Lm-crlf3* fragment 1

TCTTGGAAGGTTGCCAGCAGTCCCAAATTTGGAAGATATTCCTGGTGTGCAGTTTGAATGCTGTCTACATGCCATTGAAGCTGACTTGGTTCATGCTGTTGAGAGGGTTGGCAGTGTGTCACGACTTGGACCTGTCCAAATTTGTGCTGTTGAAGAGAAACCTGGAGCTCTGCTGGTCCATTGGCAGCAGGTAGAGACAGAGAAACCAGCAGAAATTGGCTCATTCAGGCTACAACGAGCATACGGAGATGCACGTGGCCGTCGTGAGTTAGAAGCCAATTTCCACGATGAGTATGTGGGACCTGAATGTCACCACCTTATACG

## RNAi *Lm-crlf3* fragment 2

GGAACCAGTCACTCTGCGAGTCTGTTGCCGTGAAGACAGTGAGCTTGCACCGTGGAGTACTTGGTCTGTGTTACATGTTGCTGCAACCTCACTACCTCCATTCTGCTGGGAGGGAACAAATCAGAACTACACTATAACAAATGAGAAACAACTGGCCACAAAAAGCACCGCAGAAACGTCTGTCCTTTTTTCATGTGGGCCACAATTTGGACCAGGACATGCAGTGGAATTCACTGTTCTAGAATGTGGTGCAGGATGTAGTGACGAAGGTCTGGCTCTTGCCGATCACCATTTTAGTGATGAAAATCTTCTCCAGCCTGGGGTAATATTCG

## *Lm*-CRLF3

MEQSKSNSVFVEAVDAAEEYVKKLEDLLKTLTEAEHQVKHSAKQARADVNFALDTLAATFTNALKRRRSMLLEEVDRLCSEGLEPLIECRDLITTQMQVAHSYANEGRHSLNGSNKCPVAESWDDYTHSYAECGAAFLGRLPAVPNLEDIPGVQFECCLHAIEADLVHAVERVGSVSRLGPVQICAVEEKPGALLVHWQQVETEKPAEIGSFRLQRAYGDARGRRELEANFHDEYVGPECHHLIRNLRPKEPVTLRVCCREDSELAPWSTWSVLHVAATSLPPFCWEGTNQNYTITNEKQLATKSTAETSVLFSCGPQFGPGHAVEFTVLECGAGCSDEGLALADHHFSDENLLQPGVIFVNAQGSVFVDGKGKTTKLPPLEKGSKLCFTCEHVRSSKVRIHIDSGNKTVAYDWNVSSPLQKLFFAISFGQVGWKVLVE

## *Lm*-rpt3 (Accession Number MN245517)

CDSTTGGGGATCGGTGCGTCAGATGATACAGATTCCGAAGATTTGTACACAAAGTATAAGAAACTTCAGAGGCAGCTAGAATTCTTAGCTGTGCAGGAAGAATACATTAAGGATGAGCAGCGCAATTTGAAAAAGGAATACCTGCACGCTCAGGAGGAAGTTAAGCGGATACAGAGTGTCCCCCTGGTTATCGGGCAGTTCCTCGAAGCTGTTGACCAGAATACTGGAATAGTTGGCAGCACTACAGGTTCAAACTATTATGTCAGAATACTCTCAACAATTGACCGGGAACTTCTGAAACCTTCTGCAAGTGTTGCCCTTCATAAACACAGCAATGCACTGGTTGATGTTCTTCCACCTGAGGCTGATTCATCTATATCAATGCTTCAGGCAGATGAAAAGCCAGATGTGACATACTCAGACATTGGAGGTATGGACATGCAGAAGCAAGAAATTCGTGAGGCTGTGGAGTTACCCTTGACACACTTCGAACTGTACAAACAGATCGGTATAGACCCCCCACGAGGTGTGCTGATGTATGGGCCACCAGGCTGTGGAAAGACCATGCTGGCAAAGGCTGTCGCTCACCATACTACAGCGGCTTTCATCCGTGTTGTCGGCTCAGAGTTTGTGCAGAAATATTTAGGAGAAGGTCCAAGAATGGTGAGAGATGTTTTCAGACTTGCCAAGGAAAACTCTCCCGCCATCATATTCATTGATGAGATTGATGCCATAGCAACAAAGCGATTTGATGCCCAGACTGGTGCAGACAGAGAAGTGCAAAGAATATTGTTAGAATTGCTCAACCAAATGGATGGCTTCGATCAAACAACAAATGTTAAGGTGATAATGGCAACGAATCGTGCAGACACTCTTGACCCTGCACTACTCAGGCCCGGCCGTCTTGATCGTAAGATCGAATTTCCTTTACCTGACCGGAGGCAAAAGCGGTTGATTTTCTCAACCATTACAAGCAAGATGAACCTGAGTGAAGAAGTGGACCTTGAGGATTATGTTGCTCGTCCAGATCGCATCTCTGGTGCAGACATCAATGCCATCTGTCAGGAGGCTGGAATGCATGCAGTACGTGAGAACCGCTACATAGTACTTACAAAAGATTTCGAAAAGGGATACAAAAATAATATAAAGAAGGATGAATCAGAGCATGAATTCTATAAATAA

## RNAi *Lm-rpt3* fragment

GATGAGCAGCGCAATTTGAAAAAGGAATACCTGCACGCTCAGGAGGAAGTTAAGCGGATACAGAGTGTCCCCCTGGTTATCGGGCAGTTCCTCGAAGCTGTTGACCAGAATACTGGAATAGTTGGCAGCACTACAGGTTCAAACTATTATGTCAGAATACTCTCAACAATTGACCGGGAACTTCTGAAACCTTCTGCAAGTGTTGCCCTTCATAAACACAGCAATGCACTGGTTGATGTTCTTCCACCTGAGGCTGATTCATCTATATCAATGCTTCAGGCAGATGAAAAGCCAGATGTG

## *Lm*-RPT3

MGIGASDDTDSEDLYTKYKKLQRQLEFLAVQEEYIKDEQRNLKKEYLHAQEEVKRIQSVPLVIGQFLEAVDQNTGIVGSTTGSNYYVRILSTIDRELLKPSASVALHKHSNALVDVLPPEADSSISMLQADEKPDVTYSDIGGMDMQKQEIREAVELPLTHFELYKQIGIDPPRGVLMYGPPGCGKTMLAKAVAHHTTAAFIRVVGSEFVQKYLGEGPRMVRDVFRLAKENSPAIIFIDEIDAIATKRFDAQTGADREVQRILLELLNQMDGFDQTTNVKVIMATNRADTLDPALLRPGRLDRKIEFPLPDRRQKRLIFSTITSKMNLSEEVDLEDYVARPDRISGADINAICQEAGMHAVRENRYIVLTKDFEKGYKNNIKKDESEHEFYK
