# Supplementary material for: The Orphan Cytokine Receptor CRLF3 Emerged With the Origin of the Nervous System and Is a Neuroprotective Erythropoietin Receptor in Locusts
Source: Front Mol Neurosci. 2019 Oct 11;12:251. doi: 10.3389/fnmol.2019.00251 (PMC6797617; doi:10.3389/fnmol.2019.00251)
Supplement: Supplementary file 6 [file Table_3.DOCX]

#NEXUS

begin trees;

tree tree_1 = [&R] (((Branchiostoma_belcheri:0.012325,Branchiostoma_floridae:0.017614):0.90175,((Ciona_intestinalis:1.281126,(Hydra_vulgaris:1.263472,(Exaiptasia_pallida:0.254021,(Nematostella_vectensis:0.2153,(Orbicella_faveolata:0.066114,Stylophora_pistillata:0.073016):0.282425):0.05872):0.369087):0.841556):0.1096,(Priapulus_caudatus:1.624847,((Capitella_teleta:0.907863,((Octopus_bimaculoides:0.712251,(Mizuhopecten_yessoensis:0.448395,(Crassostrea_gigas:0.065224,Crassostrea_virginica:0.059234):0.399309):0.086022):0.067423,(Lottia_gigantea:0.429117,(Pomacea_canaliculata:0.497819,(Biomphalaria_glabrata:0.174205,Aplysia_californica:0.297839):0.330974):0.217572):0.081558):0.260215):0.192004,((Centruroides_sculpturatus:0.703397,Limulus_polyphemus:0.773795):0.444865,(Daphnia_magna:3.622483,((Fopius_arisanus:1.77393,(Nilaparvata_lugens:0.836626,(Locusta_migratoria:0.666096,(Gryllus_bimaculatus:0.302877,(Blattella_germanica:0.199136,(Zootermopsis_nevadensis:0.088772,Cryptotermes_secundus:0.123073):0.105089):0.119861):0.068588):0.083773):0.315619):0.225781,(Agrilus_planipennis:2.024432,(Nicrophorus_vespilloides:0.809282,(Oryctes_borbonicus:0.680904,((Dendroctonus_ponderosae:1.333375,Tribolium_castaneum:0.407003):0.110895,(Aethina_tumida:0.444539,(Leptinotarsa_decemlineata:0.355535,Anoplophora_glabripennis:0.239411):0.168455):0.075996):0.195875):0.077035):0.206519):0.35103):0.21462):0.165737):0.182164):0.065355):0.110206):0.147376):0.420103,((Rhincodon_typus:0.109472,Callorhinchus_milii:0.116643):0.091124,((Latimeria_chalumnae:0.111016,(Lepisosteus_oculatus:0.116819,((Scleropages_formosus:0.048942,Paramormyrops_kingsleyae:0.062441):0.045352,((Clupea_harengus:0.072744,((Ictalurus_punctatus:0.118874,(Astyanax_mexicanus:0.022294,Pygocentrus_nattereri:0.029187):0.022166):0.052638,(Danio_rerio:0.029244,(Sinocyclocheilus_rhinocerous:0.018516,((Carassius_auratus:0.011403,Cyprinus_carpio:0.018447):0.002071,(Sinocyclocheilus_grahami:2.0E-6,Sinocyclocheilus_anshuiensis:0.00674):0.004504):0.004916):0.010362):0.05438):0.022335):0.020085,((Esox_lucius:0.045674,(Oncorhynchus_tshawytscha:0.202226,(Salmo_salar:0.003809,(Oncorhynchus_mykiss:2.0E-6,Oncorhynchus_kisutch:3.0E-6):0.044675):0.003784):0.025983):0.046155,(Boleophthalmus_pectinirostris:0.146015,(Hippocampus_comes:0.164321,((Takifugu_rubripes:0.04048,Tetraodon_nigroviridis:0.051786):0.056078,((Larimichthys_crocea:0.011819,Labrus_bergylta:0.046002):0.005957,(Notothenia_coriiceps:0.086417,(((Seriola_lalandi_dorsalis:2.0E-6,Seriola_dumerili:0.002274):0.014092,((Monopterus_albus:0.045216,(Anabas_testudineus:0.042363,Mastacembelus_armatus:0.039158):0.005114):0.004518,(Lates_calcarifer:0.010767,(Scophthalmus_maximus:0.061769,(Cynoglossus_semilaevis:0.407866,Paralichthys_olivaceus:0.035753):9.88E-4):0.012651):0.003041):0.004663):0.007692,((Stegastes_partitus:0.006508,(Amphiprion_ocellaris:0.007217,Acanthochromis_polyacanthus:0.006553):0.009914):0.027157,((Nothobranchius_furzeri:0.064303,(Austrofundulus_limnaeus:0.042888,Kryptolebias_marmoratus:0.041922):0.037314):0.006983,(((Oryzias_melastigma:0.04168,Oryzias_latipes:0.049342):0.057216,(Neolamprologus_brichardi:0.002271,(Pundamilia_nyererei:2.0E-6,(Maylandia_zebra:0.002272,Oreochromis_niloticus:0.002271):2.0E-6):2.0E-6):0.02886):0.014078,(Fundulus_heteroclitus:0.030712,(Cyprinodon_variegatus:0.021095,((Poecilia_formosa:0.004626,Poecilia_mexicana:0.002307):0.002329,(Poecilia_reticulata:0.032181,(Xiphophorus_maculatus:0.007016,Gambusia_affinis:0.004678):0.002347):0.002235):0.022953):0.002805):0.022665):0.002196):0.010903):0.017889):0.005173):0.00324):0.015033):0.005856):0.015233):0.060883):0.045891):0.036556):0.080159):0.155826):0.022579,(((Rana_catesbeiana:1.051232,Nanorana_parkeri:0.105402):0.075603,(Xenopus_laevis:0.04921,Xenopus_tropicalis:0.067101):0.148951):0.110001,(((Gekko_japonicus:0.059287,((Anolis_carolinensis:0.202053,Pogona_vitticeps:0.071433):0.006445,(Python_bivittatus:0.015711,(Protobothrops_mucrosquamatus:0.024825,Thamnophis_sirtalis:0.032773):0.034477):0.061864):0.019167):0.0486,((Pelodiscus_sinensis:0.046733,(Chelonia_mydas:0.012502,(Chrysemys_picta_bellii:0.005691,Terrapene_mexicana_triunguis:0.063563):0.011571):0.011074):0.052509,(((Alligator_sinensis:0.090794,Alligator_mississippiensis:0.002836):0.004289,(Crocodylus_porosus:0.008234,Gavialis_gangeticus:0.02615):0.01096):0.052136,(Acanthisitta_chloris:2.0E-6,(((Corvus_brachyrhynchos:2.0E-6,Corvus_cornix_cornix:3.0E-6):0.003022,((Parus_major:0.002352,Pseudopodoces_humilis:2.0E-6):0.004918,((Sturnus_vulgaris:0.059037,Ficedula_albicollis:0.002219):0.061663,((Lepidothrix_coronata:2.0E-6,Manacus_vitellinus:0.102002):0.034087,(Taeniopygia_guttata:3.0E-6,(Lonchura_striata_domestica:0.002232,(Zonotrichia_albicollis:2.0E-6,(Serinus_canaria:0.076226,Geospiza_fortis:0.064365):0.067466):0.037126):0.004927):0.007494):0.004819):0.004647):0.008143):0.010115,(((Limosa_lapponica_baueri:0.017919,Calidris_pugnax:0.006517):0.010765,(Amazona_aestiva:0.003979,(Nestor_notabilis:0.01794,Melopsittacus_undulatus:0.099714):2.0E-6):0.023293):0.003372,((Patagioenas_fasciata_monilis:0.022383,((Coturnix_japonica:0.010972,Numida_meleagris:0.018713):0.004038,(Meleagris_gallopavo:0.006557,(Gallus_gallus:0.011826,(Bambusicola_thoracicus:0.045424,(Colinus_virginianus:0.020823,Callipepla_squamata:2.0E-6):0.03514):0.036083):3.0E-6):0.002274):0.021859):0.004066,((Antrostomus_carolinensis:0.023943,Nipponia_nippon:0.002996):0.002826,((Pygoscelis_adeliae:2.0E-6,Aptenodytes_forsteri:2.0E-6):0.005932,((Calypte_anna:0.033423,(Haliaeetus_albicilla:2.0E-6,(Aquila_chrysaetos_canadensis:2.0E-6,(Haliaeetus_leucocephalus:3.0E-6,Egretta_garzetta:0.005926):3.0E-6):3.0E-6):0.021893):0.022221,(Podiceps_cristatus:0.021096,((Merops_nubicus:0.042599,(Anas_platyrhynchos:0.001918,Anser_cygnoides_domesticus:0.010421):0.014031):0.013191,((Phoenicopterus_ruber_ruber:3.0E-6,(((Charadrius_vociferus:0.008922,Opisthocomus_hoazin:0.008968):0.002933,(Colius_striatus:0.058057,(Picoides_pubescens:0.033135,Tyto_alba:0.006703):0.005319):2.0E-6):0.010966,(Cathartes_aura:2.0E-6,((Eurypyga_helias:0.076989,Columba_livia:0.085542):0.020338,(Fulmarus_glacialis:3.0E-6,(Chlamydotis_macqueenii:0.021779,Mesitornis_unicolor:0.022279):0.011055):0.00539):0.013729):0.013639):2.77E-4):0.007953,((Cuculus_canorus:0.034941,((Nothoprocta_perdicaria:0.011423,Tinamus_guttatus:0.007197):0.008221,(Struthio_camelus_australis:0.026345,(Dromaius_novaehollandiae:0.008913,(Apteryx_rowi:2.0E-6,Apteryx_australis_mantelli:2.0E-6):0.005978):0.006039):2.0E-6):0.04127):0.021095,(((Pterocles_gutturalis:0.025796,Chaetura_pelagica:0.090372):0.026846,(Buceros_rhinoceros_silvestris:0.055095,(Tauraco_erythrolophus:0.00895,Leptosomus_discolor:0.011861):0.002947):3.0E-6):0.022507,(Pelecanus_crispus:0.007323,(Balearica_regulorum_gibbericeps:0.022092,((Phalacrocorax_carbo:0.008919,(Phaethon_lepturus:0.014867,Gavia_stellata:0.021541):3.0E-6):0.010407,(Cariama_cristata:0.049599,(Apaloderma_vittatum:0.021372,Falco_peregrinus:0.037817):0.005137):0.018166):0.021232):2.26E-4):0.011972):0.017207):0.021657):0.015336):0.013256):3.0E-6):3.0E-6):0.031237):0.044793):0.002458):0.007389):0.009998):0.034278):0.017051):0.014623):0.03395,((Ornithorhynchus_anatinus:0.092318,(Monodelphis_domestica:0.01968,(Phascolarctos_cinereus:0.032329,Sarcophilus_harrisii:0.010005):0.004689):0.016475):0.011127,(Trichechus_manatus_latirostris:0.004814,((Orycteropus_afer_afer:0.023514,(Chrysochloris_asiatica:0.032855,Elephantulus_edwardii:0.025591):0.005868):2.0E-6,(Dasypus_novemcinctus:0.016462,((((Saimiri_boliviensis_boliviensis:0.00231,(Callithrix_jacchus:0.004626,Aotus_nancymaae:0.002309):2.0E-6):0.009325,((Homo_sapiens:3.0E-6,(Pan_troglodytes:0.161172,Pan_paniscus:2.0E-6):2.0E-6):0.002318,((Cercocebus_atys:0.002332,(Macaca_mulatta:2.0E-6,(Chlorocebus_sabaeus:0.01019,(Mandrillus_leucophaeus:0.06121,Pongo_abelii:0.08343):0.008844):2.0E-6):2.0E-6):0.00235,(Piliocolobus_tephrosceles:6.6E-5,(Colobus_angolensis_palliatus:0.058436,(Rhinopithecus_roxellana:0.054467,(Theropithecus_gelada:0.042476,(Rhinopithecus_bieti:0.028447,(Macaca_nemestrina:2.0E-6,Cebus_capucinus_imitator:0.038244):2.0E-6):0.038384):0.019692):0.03554):0.042373):0.004634):2.0E-6):0.002295):0.011721,(Galeopterus_variegatus:0.009295,((Microcebus_murinus:0.006993,(Otolemur_garnettii:0.01173,Propithecus_coquereli:0.018969):2.0E-6):0.009254,((Carlito_syrichta:0.034788,(Ochotona_princeps:0.056497,Oryctolagus_cuniculus:0.014349):0.010815):0.003618,((Marmota_marmota_marmota:0.002295,(Urocitellus_parryii:0.145943,Ictidomys_tridecemlineatus:0.002289):2.0E-6):0.006952,((Castor_canadensis:0.013852,Dipodomys_ordii:0.02362):0.002513,((Fukomys_damarensis:0.13093,(Heterocephalus_glaber:0.016987,(Chinchilla_lanigera:0.023157,(Cavia_porcellus:0.014734,Octodon_degus:0.181631):0.00467):0.002679):0.001304):0.012462,(Nannospalax_galili:0.093691,(Jaculus_jaculus:0.030842,((Rattus_norvegicus:0.023719,(Mus_caroli:3.0E-6,(AAD31759__AF120152_1_:0.00231,Mus_pahari:0.009209):0.002269):0.017235):0.005529,(Meriones_unguiculatus:0.01851,((Mesocricetus_auratus:0.006851,Cricetulus_griseus:0.121145):0.004681,(Microtus_ochrogaster:0.010893,(Peromyscus_maniculatus_bairdii:0.002281,Neotoma_lepida:0.039328):0.002316):0.001998):2.0E-6):0.003032):0.016286):0.007002):0.002352):0.002565):0.0023):0.004624):0.002319):3.0E-6):0.00229):0.002328,(((Equus_caballus:0.006923,Ceratotherium_simum_simum:0.004673):0.002322,(Eptesicus_fuscus:0.006963,(Pteropus_alecto:0.002304,Rousettus_aegyptiacus:0.011616):0.002331):2.0E-6):0.002289,(((Lipotes_vexillifer:0.00229,Orcinus_orca:2.0E-6):0.00933,(((Cervus_elaphus_hippelaphus:0.327418,Odocoileus_virginianus_texanus:3.0E-6):0.00686,(Capra_hircus:3.0E-6,Ovis_aries_musimon:0.002287):0.002281):3.0E-6,(Bubalus_bubalis:3.0E-6,(Bison_bison_bison:0.03545,(Bos_indicus:2.0E-6,Bos_taurus:2.0E-6):2.0E-6):0.004553):0.002279):0.009324):0.004611,((Erinaceus_europaeus:0.018667,((Sus_scrofa:0.009205,Tupaia_chinensis:0.033709):0.007086,(Condylura_cristata:0.016339,(Hipposideros_armiger:0.00959,Rhinolophus_sinicus:0.022961):0.002053):0.002203):0.002441):2.0E-6,((Mustela_putorius_furo:2.0E-6,(Desmodus_rotundus:0.020007,(Felis_catus:2.0E-6,(Puma_concolor:0.002307,(Panthera_pardus:3.0E-6,Panthera_tigris_altaica:0.013839):0.002304):3.0E-6):0.002064):0.007317):0.002312,(Odobenus_rosmarus_divergens:2.0E-6,(Neomonachus_schauinslandi:0.004629,((Callorhinus_ursinus:0.002316,Leptonychotes_weddellii:0.006945):2.0E-6,((Vulpes_vulpes:0.017144,Canis_lupus_familiaris:0.027142):0.01037,(((Miniopterus_natalensis:0.055954,Balaenoptera_acutorostrata_scammoni:0.023706):0.015316,(Ursus_maritimus:0.041786,(Manis_javanica:0.063135,((Sorex_araneus:0.211952,Bos_mutus:0.279182):0.192784,((Ovis_aries:0.016501,Pantholops_hodgsonii:0.064817):0.023713,(Equus_asinus:0.022737,Echinops_telfairi:0.0844):0.004711):0.021684):0.025608):0.017012):0.02332):0.017161,(Acinonyx_jubatus:0.052102,((Myotis_brandtii:0.020516,(Neophocaena_asiaeorientalis_asiaeorientalis:0.002189,Delphinapterus_leucas:0.002592):0.029592):0.030901,((Ailuropoda_melanoleuca:0.031971,Equus_przewalskii:0.036145):0.026733,(Myotis_davidii:0.015009,(Myotis_lucifugus:0.02029,(Loxodonta_africana:0.06099,(Vicugna_pacos:0.013956,(Camelus_ferus:0.023109,Camelus_bactrianus:3.0E-6):0.021151):0.107771):0.089379):0.005269):0.023013):0.019333):0.017988):0.029355):0.031561):0.046573):0.002304):2.0E-6):2.0E-6):0.004627):0.002308):3.0E-6):0.004642):0.002332):0.002307):0.002125):0.030504):0.083273):0.048511):0.05116):0.040566):0.420103);

end;
